# Exogenous electrical stimulation combined with electroactive nanopatches promotes cutaneous nerve regeneration and wound healing

**DOI:** 10.3389/fbioe.2026.1852546

**Published:** 2026-09-09

**Authors:** Chenxi Zhang, Baicheng Lu, Zhe Jin, Gaoxiang Yu, Xianting Zhou, Hongqiang Wu, Jiadong Pan, Wei Guo, Shanqing Yin

**Affiliations:** 1 Department of Hand Microsurgery and Plastic Reconstructive Surgery, Ningbo No.6 Hospital, Ningbo, China; 2 Ningbo Clinical Research Center for Orthopedics, Sports Medicine & Rehabilitation, Ningbo, China; 3 Emergency Department, Peking University People’s Hospital, Beijing, China; 4 School of Medicine, Ningbo University, Ningbo, China; 5 Department of Orthopaedics, The Second Affiliated Hospital and Yuying Children’s Hospital of Wenzhou Medical University, Wenzhou, China

**Keywords:** electrical stimulation, electroactive nano patch, electrospinning, functional regeneration, neural regeneration, wound healing

## Abstract

High-quality skin wound healing requires rapid closure, complete re-epithelialization, orderly collagen remodeling, minimal scarring, and restoration of local sensory function. Substantial evidence confirms that peripheral nerve regeneration in the skin is not a passive concomitant of repair, but a critical foundation for functional tissue regeneration. However, effective combinatorial strategies to synchronously promote nerve regeneration and wound healing via a personalized electroactive microenvironment remain an unmet clinical need, driving the development of targeted electroactive biomaterials. In this study, an electroactive polycaprolactone/carbon nanotube (PCL/CNT) nanopatch was fabricated by electrospinning. Its microstructure and electroactivity were characterized, and the biosafety and efficacy of the patch combined with exogenous electrical stimulation (ES) were evaluated. *In vitro*, PC12 cell proliferation, neurite outgrowth, and neural marker expression were quantified. *In vivo* efficacy and biosafety were assessed in a Sprague-Dawley rat full-thickness skin defect model. Compared with PCL, the PCL/CNT nanopatch exhibited uniformly aligned fibers and significantly enhanced conductivity. PCL/CNT + ES enhanced PC12 cell viability, promoted neurite outgrowth, and upregulated neural markers *in vitro*. *In vivo*, this group showed accelerated re-epithelialization, thicker granulation tissue, more organized collagen deposition, and elevated density of NF200-positive nerve fibers compared with the control, pure PCL, and PCL/CNT groups. No visceral abnormalities were detected, confirming biosafety. The “topography guidance + electrical stimulation” strategy exerted a synergistic effect on skin wound healing by enhancing peripheral nerve regeneration, coordinating tissue repair, and improving healing quality. This study presents a promising therapeutic approach for high-quality wound healing and supports the rational design of electroactive biomaterials targeting synchronous nerve regeneration and wound repair.

## 1 Introduction

Cutaneous wound repair is a highly dynamic biological process encompassing inflammatory modulation, cell migration, angiogenesis, extracellular matrix remodeling, and functional tissue regeneration (Cañedo-Dorantes and Cañedo-Ayala, 2019). Clinically, the therapeutic goal of wound care has advanced beyond superficial closure to prioritize the coordinated structural and functional tissue reconstruction—namely, the achievement of high-quality healing. High-quality cutaneous wound healing is typically defined by accelerated wound closure, complete re-epithelialization, regulated collagen deposition and alignment, minimized scarring, and restoration of sensory and barrier functions that closely mimic native skin (Cañedo-Dorantes and Cañedo-Ayala, 2019; Mathew-Steiner et al., 2021). Thus, devising strategies to accelerate wound closure while enhancing the quality of regenerated tissue has emerged as a critical challenge in the fields of regenerative medicine and cutaneous wound repair.

In recent years, the role of cutaneous peripheral nerve regeneration in wound healing has attracted growing attention. Cutaneous peripheral nerve fibers not only mediate sensory conduction but also regulate the biological behavior of keratinocytes, fibroblasts, endothelial cells, and immune cells via the release of various neurotransmitters and neurotrophic factors, thereby modulating the remodeling of the cutaneous wound microenvironment (Johnston and Miller, 2022; Nowak et al., 2021; Xing et al., 2024). Accumulating evidence indicates that cutaneous peripheral nerve regeneration is closely correlated with wound closure, angiogenesis, collagen remodeling, and functional tissue recovery (Alapure, 2020; Johnston and Miller, 2022; Kim et al., 1998; Nowak et al., 2021). Indeed, the reconstruction of cutaneous neural networks within the wound bed is not merely a concomitant event, but rather an essential driver of high-quality cutaneous wound healing (Johnston and Miller, 2022; Nowak et al., 2021). Therefore, elucidating strategies to enhance cutaneous peripheral nerve regeneration in wound tissues and utilize it as a core driver to elevate wound healing quality carries important scientific significance and considerable clinical potential.

Endogenous electric fields are widely present during cutaneous wound repair and act as key bioelectrical cues that modulate directional cell migration, proliferation, and differentiation (Nuccitelli, 2003; Zhao et al., 2010). Following cutaneous wound formation, the local electrophysiological microenvironment is perturbed, and these endogenous electrical cues are tightly correlated with re-epithelialization, tissue regeneration, and peripheral neural outgrowth (Nuccitelli, 2003; Zhao et al., 2010). Building on this premise, exogenous electrical stimulation (ES) has been increasingly utilized in cutaneous tissue healing research to mimic or potentiate physiological electrical cues, thus optimizing the cutaneous wound healing microenvironment (Cheah et al., 2021; Li et al., 2012; Zhao et al., 2010). Notably, in peripheral nerve regeneration research, ES has been shown to enhance neuronal migration, axonal outgrowth, and neural differentiation, highlighting its potential as a key regulatory strategy to couple cutaneous wound repair and peripheral nerve regeneration (Chang et al., 2013; Juckett et al., 2022; Koppes et al., 2014).

To efficiently deliver exogenous electrical cues to wound tissues, the development of scaffold materials integrating biocompatibility and electrical conductivity is critical. Electrospun scaffolds are extensively employed in tissue engineering for their extracellular matrix-mimetic architecture, high specific surface area, and superior design versatility (Jun et al., 2018). Polycaprolactone (PCL) demonstrates favorable biocompatibility and biodegradability, whereas carbon nanotubes (CNTs) feature outstanding electrical conductivity and mechanical properties (Rahimkhoei et al., 2023; Wang et al., 2020). The combination of these two materials enables the fabrication of a composite electroactive nanopatch integrating topographical guidance and electrical signal conduction capabilities, thus furnishing a tailored physical and electrical microenvironment for cutaneous wound tissue regeneration (Pi et al., 2022; Wang et al., 2020).

Building on this rationale, to facilitate high-quality cutaneous wound healing, this study fabricated a conductive PCL/CNT electroactive nanopatch and combined it with exogenous electrical stimulation (ES). We systematically evaluated its pro-regenerative effects on *ex vivo* neuronal differentiation, as well as its therapeutic efficacy in wound repair quality and peripheral nerve regeneration in an *in vivo* rat model of full-thickness cutaneous skin defects. We focused on key outcome measures including wound closure, re-epithelialization, granulation tissue formation, collagen deposition and alignment, and cutaneous peripheral nerve fiber regeneration within the wound bed. The overarching objective of this work is to achieve faster, more effective, and functional high-quality cutaneous wound healing by developing a combinatorial therapeutic strategy that leverages an electroactive microenvironment to enhance cutaneous peripheral nerve regeneration.

## 2 Materials and methods

### 2.1 Preparation and characterization of PCL/CNT nanopatches

Polycaprolactone (PCL) was purchased from Sigma-Aldrich (St. Louis, MO, United States). Trifluoroethanol (TFE) was obtained from Macklin Biochemical Co., Ltd. (Shanghai, China). Carboxyl-functionalized multi-walled carbon nanotubes (MWCNT-COOH) were purchased from XFNANO Co., Ltd. (Nanjing, China).

For the fabrication of pure PCL nanopatches, 1 g of PCL was first dissolved in 10 mL of TFE to prepare the electrospinning solution. The homogeneous solution was loaded into a syringe pump, and electrospinning was conducted under the following parameters: applied voltage of 15 kV, flow rate of 1 mL/h, collector drum rotation speed of 1800 rpm, and working distance of 15 cm. The as-spun PCL nanopatches were collected and subsequently vacuum-dried overnight at room temperature for further use. For PCL/CNT composite nanopatches, 1 g of PCL was dissolved in 8 mL of TFE, while 0.03 g of MWCNT-COOH was separately dispersed in 2 mL of TFE under homogenization. The MWCNT-COOH content was 3 wt% relative to the PCL mass, corresponding to approximately 2.91 wt% of the total PCL/CNT solid content in the final electrospun nanopatch. The two solutions were then mixed uniformly to form the composite electrospinning solution, which was subjected to electrospinning under the identical parameters as those for pure PCL. The resulting PCL/CNT nanopatches were collected and vacuum-dried overnight at room temperature.

For scanning electron microscopy (SEM) characterization, the nanopatches were affixed to sample stages with conductive adhesive and sputter-coated with gold prior to observation. The microstructural morphology of the samples was examined using a field-emission SEM (Hitachi SU8010, Tokyo, Japan) at an accelerating voltage of 5 kV. The fiber diameters of PCL and PCL/CNT-PDA nanopatches were quantified by analyzing SEM micrographs using Image-Pro Plus 6.0 software (Media Cybernetics, Rockville, MD, United States). Transmission electron microscopy (TEM, JEM-1400PLUS, JEOL, Tokyo, Japan) was further employed to characterize the internal microstructure of the PCL/CNT nanopatches.

The electrical conductivity of pure PCL and PCL/CNT nanopatches was measured using a four-point probe system (OCA20, DataPhysics Instruments, Filderstadt, Germany) at room temperature.

### 2.2 Cell viability assay

PC12 cells were used for all *in vitro* cellular experiments, with three experimental groups designed: pure PCL, PCL/CNT, and PCL/CNT + exogenous electrical stimulation (ES). Exogenous ES was applied at an intensity of 100 mV/cm for 10 min daily for the corresponding group (Oprea et al., 2022). This low-intensity stimulation protocol was selected based on previous studies and was intended to provide a mild bioelectrical cue while maintaining cell viability.

PC12 cells were seeded onto the respective nanopatches of the pure PCL, PCL/CNT, and PCL/CNT + ES groups at a density of 5 × 10^4^ cells/mL. At 1, 3, and 5 days post-seeding, the samples were gently rinsed with phosphate-buffered saline (PBS), followed by incubation with a 10% (v/v) CCK-8 solution (Dojindo Laboratories, Tokyo, Japan). After a 2 h incubation at 37 °C in a humidified atmosphere with 5% CO_2_, the absorbance of each well was measured at a wavelength of 450 nm using a microplate reader (SpectraMax M2, Molecular Devices, Sunnyvale, CA, United States).

### 2.3 Live/dead staining assay

To further evaluate the *in vitro* biosafety of the nanopatches, PC12 cells were co-cultured with the respective pure PCL, PCL/CNT, and PCL/CNT + ES nanopatches for 1, 3, and 5 days using the identical experimental procedure described above. Subsequently, the samples were gently rinsed with phosphate-buffered saline (PBS) to remove non-adherent cells, and stained with a calcein-acetoxymethyl ester (calcein-AM)/propidium iodide (PI) dual-staining solution for 30 min at 37 °C in the dark. After thorough rinsing with PBS to eliminate excess staining solution, cell viability and live/dead cell distribution were observed and imaged using a confocal laser scanning microscope (CLSM, Olympus FV1000, Tokyo, Japan).

### 2.4 Cell morphology and differentiation assay

To assess the effects of the nanopatches on the differentiation, morphological polarization, and neurite outgrowth of PC12 cells, the cells were co-cultured with the respective nanopatches for 3 days following the identical experimental protocol described previously. Subsequently, the adherent cells were stained with Alexa Fluor™ 594/488-conjugated phalloidin (Abcam, Cambridge, United Kingdom). Cell morphology, cellular orientation, and neurite outgrowth length were then examined and captured via CLSM. For quantitative analysis of cellular alignment, a line was manually drawn along the longest axis of each individual cell using ImageJ software (National Institutes of Health, Bethesda, MD, United States), and the deviation angle between each cell and the mean alignment direction of the nanofibers within the nanopatch was calculated.

### 2.5 Quantitative real-time polymerase chain reaction (qRT-PCR)

After 3 days of co-culture with the respective nanopatches, total RNA was extracted from adherent PC12 cells using an RNA purification kit (ES Science, Shanghai, China). Complementary DNA (cDNA) was synthesized from the extracted total RNA using a reverse transcription kit (ABM, Vancouver, BC, Canada). Subsequently, qRT-PCR assays were performed on a CFX96™ Real-Time PCR Detection System (Bio-Rad Laboratories, Hercules, CA, United States) with SYBR Green Real-Time PCR Master Mix (TOYOBO Co., Ltd., Osaka, Japan). The relative expression levels of the NF200, GAP43, and Tubb3 genes were calculated via the 2^−ΔΔCt^ method and normalized to the endogenous reference gene GAPDH. The primer sequences used in this study were as follows: GAPDH - Forward primer (FP): GAA​GGG​CAT​CTT​GGG​CTA​CAC; Reverse primer (RP): GTT​GTC​ATT​GAG​AGC​AAT​GCC​A; NF200 - FP: ACA​TGG​CCT​CCT​ACC​AGG​AT; RP: TCT​TGA​CGT​TGA​GCA​GGT​CC; GAP43 - FP: GGA​GAA​GGA​TGA​TGC​TCC​CG; RP: TCG​CCC​TTC​TTC​TCC​TCA​GA; Tubb3 - FP: GGG​CCA​AGT​TCT​GGG​AAG​TC; RP: AGT​CGC​CCA​CGT​AGT​TGC​C. Each experimental group included at least three independent biological replicates to ensure the reliability and reproducibility of experimental data.

### 2.6 Full-thickness skin defect model in SD rats

All animal experimental procedures were performed in accordance with relevant animal ethical guidelines and approved by the Animal Ethics Committee of Peking University People’s Hospital (Approval No. 2026PHE088). Healthy adult Sprague-Dawley (SD) rats with a body weight of approximately 200 ± 20 g were selected to establish a dorsal full-thickness cutaneous defect model, for the purpose of verifying the pro-regenerative effects of exogenous electrical stimulation (ES) combined with the conductive electrospun PCL/CNT nanopatch on cutaneous tissue regeneration. The experimental animals were randomly assigned to four experimental groups (n = 10 per group): an untreated control group, a PCL group, a PCL/CNT group, and a PCL/CNT + ES group. All rats were anesthetized with 5% isoflurane (RWD Life Science, Shenzhen, China) inhalation for 5 min for induction, followed by maintenance under 3% isoflurane during the surgical procedures. After anesthesia, the dorsal hair of rats was shaved and the skin was disinfected with a sterile disinfectant. A circular full-thickness cutaneous defect with a diameter of 1 cm was created along the dorsal midline using a skin biopsy punch. With the exception of the untreated control group, the wound sites of rats in the other three groups were covered with the corresponding nanopatches and secured with sterile adhesive dressings. Specifically, the PCL group received a pure PCL nanopatch, the PCL/CNT group a PCL/CNT nanopatch, and the PCL/CNT + ES group a PCL/CNT nanopatch with the subsequent application of exogenous ES. For the PCL/CNT + ES group, ES was delivered via a local direct current (DC) electric field at an intensity of 100 mV/cm for 10 min daily. The same stimulation parameters were used *in vivo* to ensure consistency with the *in vitro* protocol and to avoid excessive electrical or thermal stimulation of the wound tissue.

To monitor the cutaneous wound healing process, digital photographs of the wound sites were captured on the day of surgery (designated as day 0) and on postoperative days (POD) 3, 7, and 14. Wound areas were quantified via professional image analysis software, and the wound healing rate was calculated using the following formula:

Healing rate (%) = [(A_0_ - A_t_)/A_0_] × 100%

Where A_0_ is the initial wound area on day 0; A_t_ is the wound area at the specific postoperative time point.

### 2.7 Histological analysis

On postoperative days (POD) 7 and 14, five rats per group (50% of each group) were euthanized using carbon dioxide (CO_2_) at a displacement rate of 50% of the chamber volume per minute for 10.5 min to ensure irreversible euthanasia. The cutaneous wound sites with adjacent granulation tissue were carefully harvested *en bloc*. The harvested tissue samples were fixed in 4% (w/v) paraformaldehyde solution for 24 h at room temperature, dehydrated through a graded ethanol series, routinely embedded in paraffin, and sectioned into 5 μm-thick serial sections. The sections were stained with hematoxylin and eosin (H&E) and Masson’s trichrome stain to observe the histological morphology of wound tissue and collagen deposition as well as alignment, thus enabling a comprehensive assessment of cutaneous wound repair efficacy. Additionally, immunofluorescence staining was performed on the wound tissue sections at POD 14. The paraffin sections were first deparaffinized, rehydrated, and subjected to antigen retrieval; subsequent to non-specific binding blocking, the sections were incubated with a primary anti-NF200 antibody to label newly formed cutaneous peripheral nerve fibers. Cell nuclei were counterstained with 4′,6-diamidino-2-phenylindole (DAPI) to visualize tissue structure, thereby quantitatively and qualitatively evaluating cutaneous peripheral nerve regeneration within the wound bed.

For the assessment of systemic biocompatibility of nanopatch implantation, major visceral organs (heart, liver, spleen, lung, and kidney) were collected from rats in the control and PCL/CNT groups at euthanasia on POD 14. The organ tissues were processed into paraffin sections following the identical fixation, embedding and sectioning protocol described above, and stained with H&E. Pathological changes in the organ tissues were observed under a light microscope to evaluate potential systemic toxic effects induced by the PCL/CNT nanopatch.

### 2.8 Statistical analysis

All of the numerical data were presented as mean ± standard deviation. Differences between two groups were analyzed using Student’s t-test, and differences between multiple groups were analyzed using one-way analysis of variance (ANOVA) fol-lowed by Tukey’s post-hoc test. All analyses were performed using SPSS 22.0 software (IBM SPSS, Chicago, IL, United States). The statistical tests conducted in this study are also represented by legends: *p < 0.05, **p < 0.01.

## 3 Results

### 3.1 Characterization of the electroactive PCL/CNT nanopatches

**FIGURE 1:**
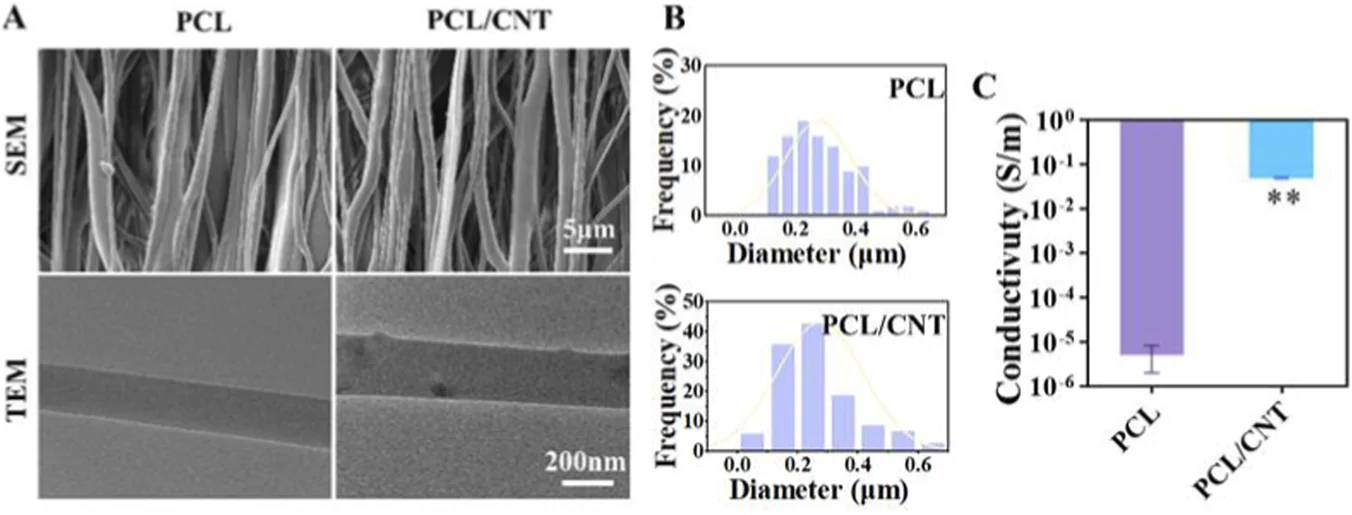
**(A)** SEM and TEM images of pure PCL and PCL/CNT nanopatches. **(B)** Fiber diameter distribution of pure PCL and PCL/CNT nanopatches (n = 100). **(C)** Electrical conductivity of pure PCL and PCL/CNT nanopatches (n = 3, P < 0.01).

Scanning electron microscopy (SEM) micrographs of the electroactive nanopatches revealed that both pure PCL and PCL/CNT composite nanofibers featured smooth surfaces, uniform diameters, and a well-aligned, highly oriented nanofibrous architecture. Transmission electron microscopy (TEM) further demonstrated the presence of dark particulate aggregates and protrusions within the PCL/CNT nanofibers, whereas pure PCL nanofibers presented a homogeneous structure without any particulate inclusions (Figure 1A). The fiber diameter distribution analysis (Figure 1B) showed that the average diameter of pure PCL nanofibers was 0.2788 ± 0.1099 μm, while that of PCL/CNT nanofibers was 0.2764 ± 0.1538 μm, with no statistically significant difference observed between the two groups (P > 0.05). Four-point probe conductivity measurements (Figure 1C) indicated that the average electrical conductivity of pure PCL nanopatches was (5.117 ± 3.124) × 10^−6^ S/cm, in contrast to (5.044 ± 0.2137) × 10^−2^ S/cm for PCL/CNT nanopatches. The electrical conductivity of the PCL/CNT composite nanopatches was significantly higher than that of pure PCL nanopatches (P < 0.01).

### 3.2 Cell viability and biocompatibility

**FIGURE 2:**
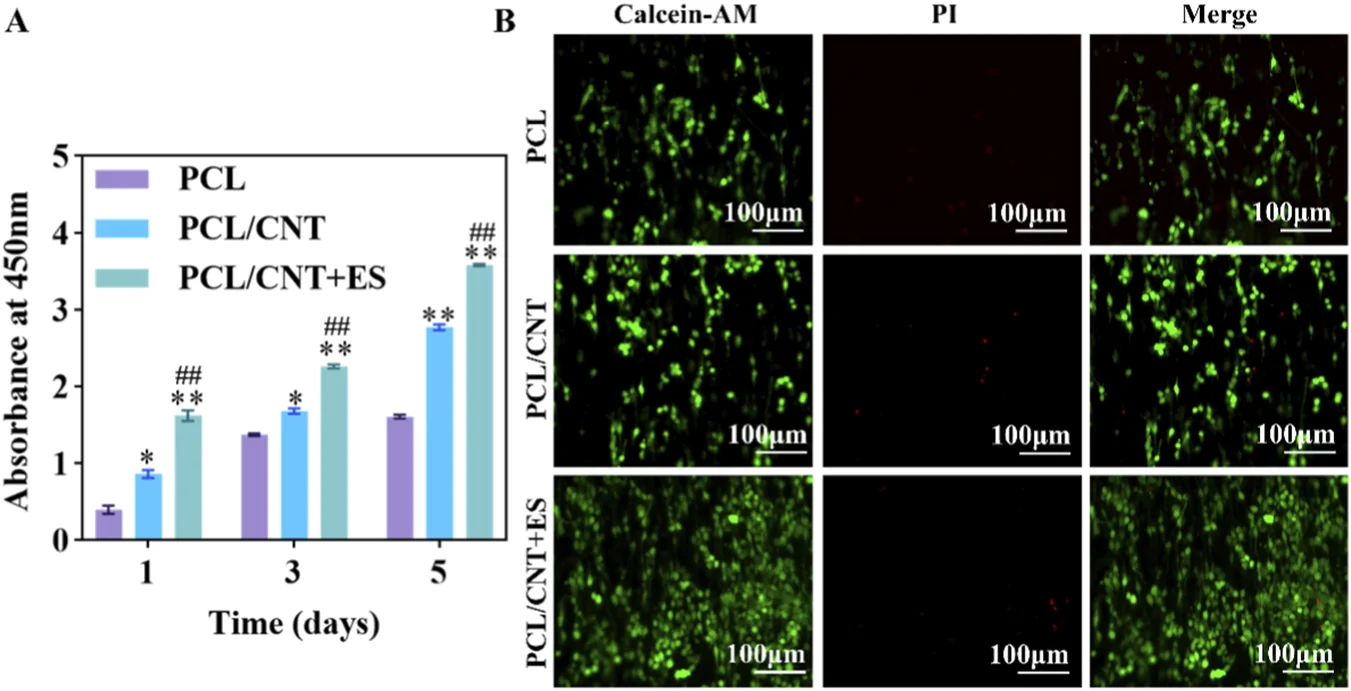
**(A)** Cell viability of PC12 cells in the pure PCL, PCL/CNT, and PCL/CNT + ES groups (n = 3, *P < 0.05, **P < 0.01 vs. pure PCL group; ##P < 0.01 vs. PCL/CNT group). **(B)** Live/dead staining images of PC12 cells in the pure PCL, PCL/CNT, and PCL/CNT + ES groups.

The CCK-8 assay results (Figure 2A) demonstrated that the PCL/CNT and PCL/CNT + ES nanopatches exerted a sustained proliferative-promoting effect on PC12 cell proliferation, in comparison to the pure PCL nanopatch group. At 1 day of culture, the cell viability in the PCL/CNT group was significantly higher than that in the pure PCL group (P < 0.05). With the extension of the culture period to 3 and 5 days, the PCL/CNT + ES group exhibited markedly enhanced proliferative-promoting activity, and its cell viability was further significantly elevated compared with the PCL/CNT group (P < 0.01).

Live/dead cell staining results (Figure 2B) further confirmed that the PCL/CNT + ES group showed intense green fluorescent signals (indicating viable cells), with a cell density significantly higher than that of the pure PCL and PCL/CNT groups (P < 0.01). In contrast, red fluorescent signals (indicating dead cells) were only sporadically detected across all three groups. These live/dead staining observations were highly consistent with the cell proliferation data obtained from the CCK-8 assay.

### 3.3 Morphological observation of neural differentiation

**FIGURE 3:**
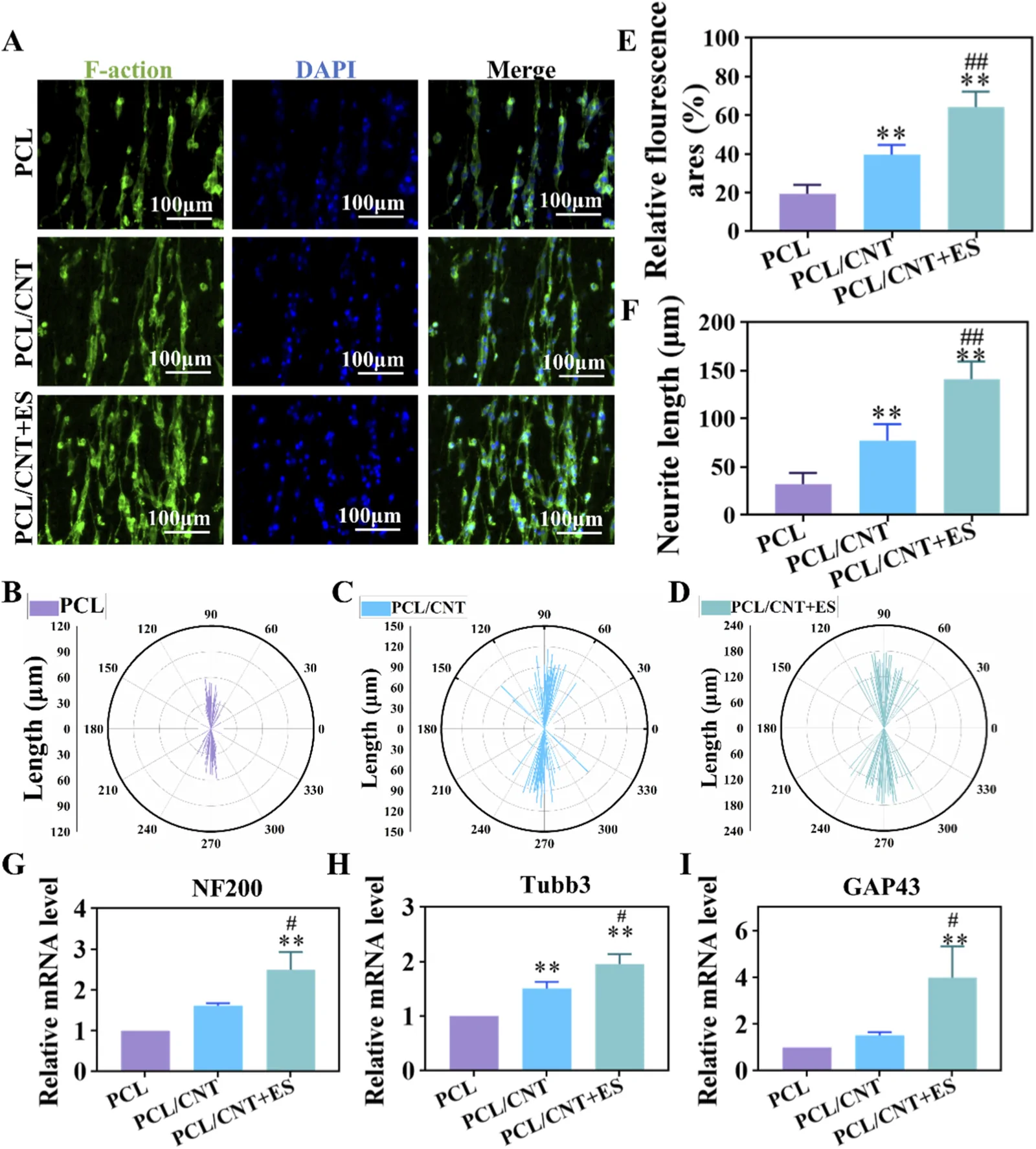
**(A)** Fluorescence images of PC12 cells stained with phalloidin-Alexa Fluor 594/488 (Abcam) in the pure PCL, PCL/CNT, and PCL/CNT + ES groups. **(B)** Axon length and orientation of PC12 cells in the pure PCL group. **(C)** Axon length and orientation of PC12 cells in the PCL/CNT group. **(D)** Axon length and orientation of PC12 cells in the PCL/CNT + ES group. **(E)** Relative fluorescence area of PC12 cells in the pure PCL, PCL/CNT, and PCL/CNT + ES groups (n = 6). **(F)** Axon length of PC12 cells in the pure PCL, PCL/CNT, and PCL/CNT + ES groups (n = 50). **(G-I)** Relative mRNA expression levels of NF200, Tubb3, and GAP43 in PC12 cells from the pure PCL, PCL/CNT, and PCL/CNT + ES groups (n = 3; *P < 0.05, **P < 0.01 vs. pure PCL group; #P < 0.05 vs. PCL/CNT group).

As shown in Figure 3A, PC12 cells in all three groups displayed typical neuron-like morphological characteristics to varying extents after 3 days of culture, which were characterized by elongated axonal outgrowths and branched neuritic structures. All three groups exhibited robust proliferative and differentiative activity. Quantitative morphological analysis demonstrated that, relative to the pure PCL group, both the PCL/CNT and PCL/CNT + ES groups showed a significant increase in cell adhesion density, accompanied by a gradient expansion in cell spreading area. Specifically, the cell coverage area in the PCL/CNT group was approximately 1.8-fold that of the pure PCL group, while the PCL/CNT + ES group further presented a cell coverage area more than 1.6-fold that of the PCL/CNT group. Furthermore, morphological observations (Figures 3B–D) revealed that cells in all three groups exhibited well-aligned orientation consistent with the nanofibrous direction of the nanopatches. Notably, quantitative analysis of neurite outgrowth (Figures 3E,F) showed that the total lengths of axonal and branched neuritic outgrowths in PCL/CNT group PC12 cells (77.13 ± 17.05 μm) were significantly longer than those in the pure PCL group (32.10 ± 11.73 μm), a 2.4-fold increase relative to the latter. The PCL/CNT + ES group exhibited a further marked elevation in neurite outgrowth length (141.1 ± 18.03 μm) compared with the PCL/CNT group, which represented a 4.4-fold increase relative to the pure PCL group.

### 3.4 Expression of neural differentiation-related genes

qRT-PCR analysis results (Figures 3G–I) demonstrated that the expression of neural differentiation-related genes was significantly upregulated in the PCL/CNT and PCL/CNT + ES groups relative to the pure PCL group. For NF200 (Figure 3G), its expression in the PCL/CNT group was 1.62-fold higher than that in the pure PCL group, while the PCL/CNT + ES group showed a more pronounced upregulation, with the expression level reaching 2.49-fold that of the pure PCL group. With respect to GAP43 (Figure 3H), the PCL/CNT group exhibited an approximate 1.51-fold upregulation of this gene relative to the pure PCL group, and the PCL/CNT + ES group further achieved a 3.98-fold upregulation compared with the pure PCL group. For Tubb3 (Figure 3I), a key neuronal maturation marker, both the PCL/CNT and PCL/CNT + ES groups induced a sustained upregulation of its expression relative to the pure PCL group. The expression level of Tubb3 in the PCL/CNT + ES group was 1.96-fold that of the pure PCL group, a significant increase compared with the 1.50-fold upregulation observed in the PCL/CNT group.

### 3.5 Assessment of wound healing efficacy

**FIGURE 4:**
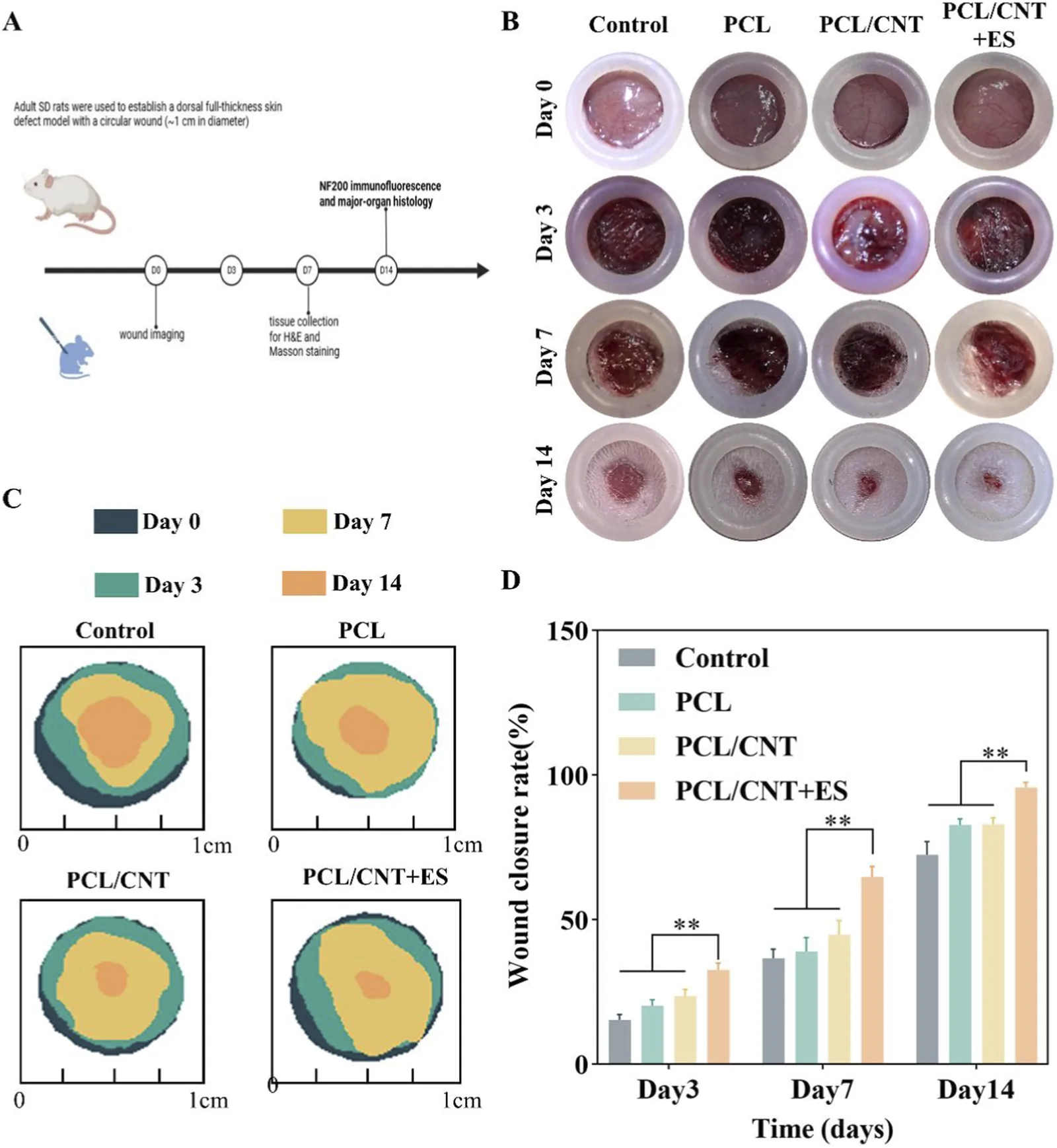
**(A)** Schematic diagram of the animal experimental protocol. **(B)** Representative macroscopic photographs of cutaneous wounds in the Control, pure PCL, PCL/CNT, and PCL/CNT + ES groups on postoperative days (POD) 0, 3, 7, and 14. **(C)** Schematic overlay of wound boundaries at different time points (scale bar, 1 cm). **(D)** Wound closure rate of each group (n = 5, *P < 0.05, **P < 0.01).

In the rat full-thickness cutaneous defect model, wound healing processes across all experimental groups were compared to further validate the pro-healing and pro-neuroregenerative effects of the electroactive PCL/CNT nanopatches combined with exogenous electrical stimulation (ES) on cutaneous tissue repair and peripheral nerve regeneration. The animal experimental protocol is schematically illustrated in Figure 4A. Macroscopic wound healing observations (Figure 4B) revealed that the cutaneous wound area in all groups gradually diminished over the experimental period, with notable differences in wound healing rates observed among the groups. At POD 7, the PCL/CNT + ES group exhibited significantly accelerated wound contraction compared with the pure PCL and PCL/CNT groups. As shown in Figures 4C,D, at POD 14, wounds in the PCL/CNT + ES group achieved nearly complete closure, with the defect area largely filled with newly formed granulation tissue, whereas distinct unhealed residual areas were still evident in both the pure PCL and PCL/CNT groups. Quantitative analysis demonstrated that the wound closure rate of the PCL/CNT + ES group reached 95.68% ± 1.68% at POD 14, which was significantly higher than those of the pure PCL group (82.81% ± 1.82%) and the PCL/CNT group (82.99% ± 1.96%) (P < 0.01).

### 3.6 Histological analysis

**FIGURE 5:**
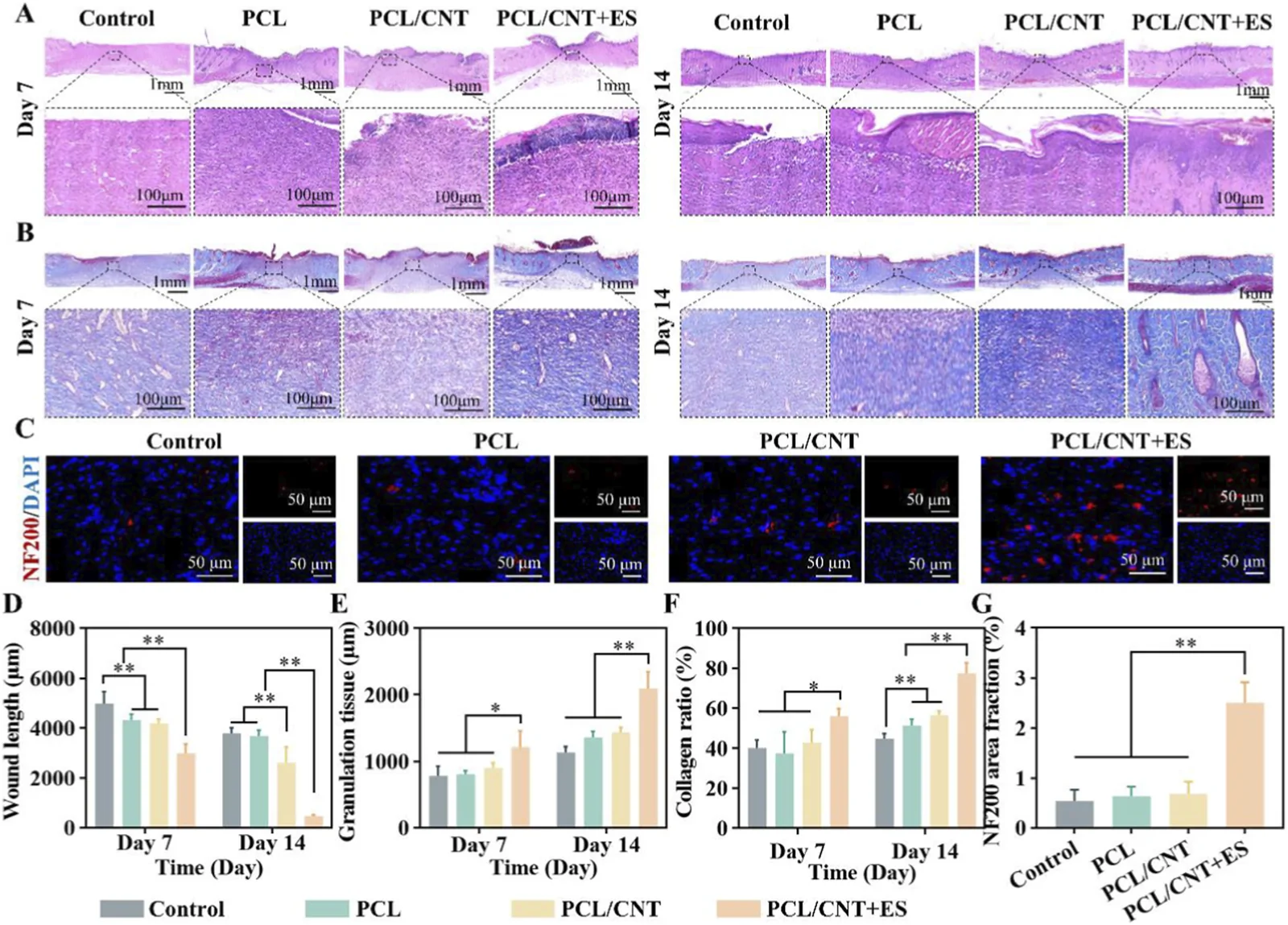
**(A)** Representative H&E staining images of cutaneous wound tissues at postoperative days 7 and 14 (top: low-magnification full-thickness view; bottom: corresponding high-magnification view), showing re-epithelialization and granulation tissue formation. Scale bars: 1 mm (low magnification), 100 μm (high magnification). **(B)** Representative Masson’s trichrome staining images of cutaneous wound tissues at POD 7 and 14 (collagen fibers stained blue), for assessing collagen deposition and arrangement. Scale bars as in **(A)**. **(C)** Representative immunofluorescence images of cutaneous wound tissues at POD 14 for NF200 (NF200, red; DAPI, blue), for evaluating newly formed peripheral nerve fibers. Scale bar: 50 μm. **(D)** Quantitative analysis of scar width (μm); **(E)** Quantitative analysis of newly formed granulation tissue thickness (μm); **(F)** Quantitative analysis of collagen-positive area percentage (%); **(G)** Quantitative analysis of NF200-positive area percentage (%) (n = 5).

Hematoxylin and eosin (H&E) staining results (Figure 5A) further elucidated the differences in cutaneous wound tissue healing quality among the groups. At POD 7, wounds in the PCL/CNT + ES group had formed relatively thick and vascularized granulation tissue, with partial epidermal coverage observed in localized areas. In contrast, the granulation tissue in the pure PCL and PCL/CNT groups was relatively loose, thin, and accompanied by limited epidermal re-epithelialization. By POD 14, the epidermis of wounds in the PCL/CNT + ES group was continuous and intact, with a well-organized dermal architecture characterized by dense cellular infiltration and collagen deposition. In comparison, the pure PCL and PCL/CNT groups still exhibited epidermal defects, with prominent wide scar tissue remaining in the wound bed. Quantitative analysis revealed that the scar width in the PCL/CNT + ES group at POD 14 was 468.23 ± 57.40 μm, which was significantly smaller than that in the pure PCL group (3691.18 ± 232.70 μm) and the PCL/CNT group (2615.11 ± 638.65 μm) (P < 0.01) (Figure 5D). Furthermore, the granulation tissue thickness in the PCL/CNT + ES group reached 2093.57 ± 250.03 μm, which was greater than that in the pure PCL group (1360.33 ± 91.92 μm) and the PCL/CNT group (1439.14 ± 75.96 μm) (Figure 5E). These results indicate that the PCL/CNT + ES group achieved superior cutaneous wound repair quality compared to the other groups, characterized by reduced scar formation and more substantial, well-developed granulation tissue.

Masson’s trichrome staining was concurrently employed to evaluate collagen deposition and remodeling within the wound area (Figure 5B). The results demonstrated that by POD 14, the PCL/CNT + ES group wounds exhibited abundant newly synthesized collagen fibers, with collagen bundles densely packed and well-aligned. Conversely, only sparse and disorganized collagen deposition was observed in the pure PCL and PCL/CNT groups. Quantitative analysis of collagen content confirmed that the collagen-positive area ratio in the PCL/CNT + ES group was significantly higher than that in the pure PCL and PCL/CNT groups (P < 0.01) (Figure 5F). This finding suggests that the electroactive PCL/CNT nanopatches combined with exogenous electrical stimulation (ES) effectively promote collagen synthesis and ordered remodeling in wound tissue, thereby enhancing the mechanical strength and structural stability of the newly formed cutaneous tissue.

To assess cutaneous peripheral nerve regeneration within the wound bed, NF200/DAPI immunofluorescence staining was performed on wound tissues harvested at POD 14 (Figure 5C). The results revealed numerous bright NF200-positive nerve fibers (green fluorescence) penetrating the newly formed granulation tissue in the PCL/CNT + ES group, with cell nuclei counterstained blue by DAPI, forming an interconnected network of regenerated peripheral nerve fibers. In contrast, only sporadic and scarce NF200-positive signals were detected in the pure PCL and PCL/CNT groups. Quantitative analysis (Figure 5G) showed that the NF200-positive area ratio in the PCL/CNT + ES group wound tissue was 2.51 ± 0.41 %Area, which was significantly higher than that in the pure PCL group (0.63 ± 0.19 %Area) and the PCL/CNT group (0.68 ± 0.25 %Area) (P < 0.001).

**FIGURE 6:**
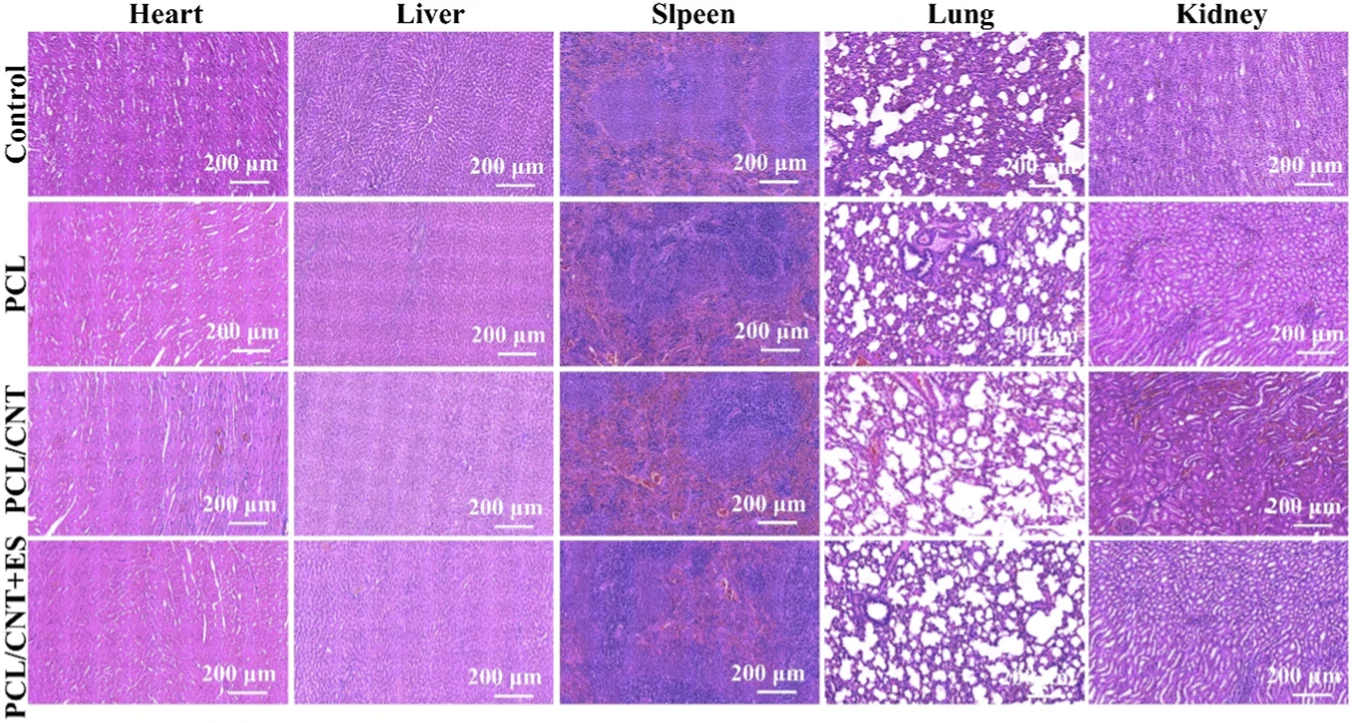
Hematoxylin and eosin (H&E) staining images of major visceral organs (heart, liver, spleen, lung, and kidney) harvested from rats at postoperative days 14. Scale bar: 200 μm.

Lastly, hematoxylin and eosin (H&E) staining was performed on the major visceral organs (heart, liver, spleen, lung, and kidney) of rats from all experimental groups (Control, pure PCL, PCL/CNT, and PCL/CNT + ES) at POD 14 (Figure 6) to evaluate the *in vivo* biosafety of the implanted electroactive PCL/CNT nanopatches and ES. The results demonstrated that no obvious inflammatory cell infiltration, structural damage, or abnormal pathological changes were observed in the visceral organ sections of any group. The histological architecture and morphology of all organ tissues were consistent with those of normal rat visceral tissues, with no evident toxic damage induced by the PCL/CNT nanopatches or exogenous ES intervention.

## 4 Discussion

Evaluative criteria for cutaneous wound repair have shifted from a sole focus on wound closure to an emphasis on high-quality healing (Cañedo-Dorantes and Cañedo-Ayala, 2019; Mathew-Steiner et al., 2021). Unlike the simple reduction of wound size, high-quality healing prioritizes the restoration of structural integrity, mechanical properties, and physiological function in newly formed cutaneous tissue. This entails continuous and complete epidermal re-epithelialization, well-regulated granulation tissue formation, orderly collagen fiber remodeling, scar minimization, and the recovery of sensory function. In this reparative process, the critical role of peripheral nerve regeneration within the wound bed has garnered growing attention. Peripheral nerve fibers in the wound bed not only mediate sensory conduction but also modulate cell migration, inflammatory responses, angiogenesis, and tissue remodeling by regulating the local wound microenvironment (Johnston and Miller, 2022; Nowak et al., 2021; Xing et al., 2024). Accordingly, promoting peripheral nerve regeneration within the cutaneous wound bed represents a pivotal strategy to improve both the rate and quality of cutaneous wound healing.

ES and electroactive biomaterials provide promising technological avenues for attaining this objective of high-quality cutaneous wound healing (Cheah et al., 2021; Nuccitelli, 2003; Zhao et al., 2010). On one hand, exogenous ES can recapitulate the physiological electrical signals inherent to injured cutaneous tissues, modulating the biological behaviors of diverse wound repair-associated cells (Cheah et al., 2021; Nuccitelli, 2003; Zhao et al., 2010). On the other hand, electroactive nanopatches act as localized carriers for the transmission and amplification of electrical signals, thereby fostering the establishment of a stable, sustainable electroactive microenvironment within the wound bed. The present study was designed and performed against this research backdrop. Its core objective is not merely to verify that electroactive nanopatches can enhance neuronal differentiation, but also to further elucidate that enhancing peripheral nerve regeneration within cutaneous wound tissue can effectively drive accelerated wound closure, orderly tissue remodeling, and sensory functional recovery—ultimately propelling cutaneous wound repair toward achieving high-quality healing.

In the present study, the ES intensity of 100 mV/cm and the stimulation duration of 10 min/day were selected to provide a low-intensity bioelectrical cue approximating the endogenous electric-field environment of injured tissues. Such mild stimulation is expected to regulate cell proliferation, migration, and neurite outgrowth while avoiding excessive electrochemical, thermal, or cytotoxic effects. The brief daily stimulation duration was designed to provide repeated regulatory signals during the wound repair process while maintaining good cell viability and tissue tolerance.

The PCL/CNT composite nanofibrous nanopatch employed in the present study integrates the excellent biocompatibility of PCL with the high electrical conductivity of CNTs. SEM and TEM characterization results demonstrated that the PCL/CNT nanofibers featured a smooth surface, a uniform diameter of 0.2764 ± 0.1538 μm, and an aligned oriented nanofibrous structure that mimics the topological characteristics of the natural peripheral nerve extracellular matrix. Notably, the incorporation of CNTs markedly enhanced the electrical conductivity of the nanopatch, which reached (5.044 ± 0.2137) × 10^−2^ S/cm—representing an approximate four-order-of-magnitude increase relative to pure PCL nanopatches (5.117 × 10^−6^ S/cm) (P < 0.01). This unique electroconductive property enables the PCL/CNT nanopatches to efficiently transduce and transmit exogenous electrical signals, thereby laying a solid foundation for modulating the electroactive microenvironment of neural cells within the wound bed.

PC12 cells, a classic cell model for neuronal differentiation, are widely used to evaluate the neurocompatibility of electroactive biomaterials. CCK-8 is a commonly used cell proliferation and cytotoxicity assay based on WST-8, a water-soluble tetrazolium salt. Its underlying principle is that dehydrogenases in the mitochondria of viable cells reduce WST-8 to an orange-yellow, water-soluble formazan product; the yield of this formazan is directly proportional to both the number of viable cells and their metabolic activity. Cell viability is thus quantifiable by measuring the optical density (OD) absorbance at a 450 nm wavelength. This assay is extensively utilized for material biocompatibility evaluation, drug toxicity screening, and cell proliferation kinetic studies, owing to its high sensitivity, operational simplicity, and a broad linear detection range In the present study, CCK-8 assay results showed that the relative proliferation rate of PC12 cells on PCL/CNT nanopatches remained consistently above 90% across all culture time points. Moreover, at 5 days of culture, the proliferative activity of cells on PCL/CNT nanopatches was significantly enhanced compared with those on pure PCL nanopatches, indicating that the PCL/CNT composite nanopatches not only exhibit no cytotoxicity but also modulate cellular metabolic activity through their intrinsic electroactive interface. Live/dead cell staining assays further validated these findings, revealing a higher density of viable cells on PCL/CNT nanopatches relative to pure PCL nanopatches, with a persistently low proportion of dead cells across all experimental groups. It is worth noting that while pristine carbon nanotubes (CNTs) may induce cytotoxicity when used alone, primarily due to surface charge accumulation or aggregation effects, their incorporation into the PCL nanofibrous matrix effectively mitigates the risk of direct CNT exposure to cells. Encapsulation within the biodegradable PCL nanofibrous matrix allows the PCL/CNT composite nanopatches to retain their core function of guiding neurite outgrowth, while simultaneously providing a critical safety guarantee for the clinical translation of this electroactive wound repair material.

Dynamic morphological changes in neuronal differentiation serve as a critical criterion for evaluating the functionality of neural repair materials, with their core essence lying in whether the regenerative niche can effectively guide the directional extension of neuronal axons and facilitate neural network reconstruction. In the present study, the growth characteristics of PC12 cells were regulated by constructing aligned PCL/CNT composite nanofibrous nanopatches. With the combined intervention of exogenous electrical stimulation (ES), the PCL/CNT + ES group exhibited a synergistic pro-differentiation effect: the axon length reached 141.1 ± 18.03 μm, representing 4.4-fold and 1.8-fold increases compared to the pure PCL group (32.10 ± 11.73 μm) and PCL/CNT group (77.13 ± 17.05 μm), respectively (P < 0.01). This synergistic enhancement originates from the integrated effects of topological guidance provided by aligned nanofibers and bioelectrical properties of the CNT conductive network. Although the present study did not directly verify the downstream signaling pathways involved, previous studies have suggested that ES may regulate neuronal differentiation through transmembrane voltage-gated calcium channels, intracellular Ca^2+^ signaling, and downstream MAPK/ERK or PI3K/Akt-related pathways. Therefore, we speculate that similar bioelectrical signaling may contribute to the enhanced neuronal polarization and neurite outgrowth observed in the PCL/CNT + ES group (Chang et al., 2013; Liu et al., 2023; Yan et al., 2014). Specifically, the physical guidance of aligned nanofibers regulates cytoskeletal reorganization via mechanotransduction, while the bioelectrical properties of the CNT conductive network transmit electrical stimuli to activate VGCCs; these two cues collaboratively promote neuronal adhesion, spreading, and directional axon outgrowth (Koppes et al., 2014). Molecular-level mechanistic investigations revealed significant differences in the expression of neuron-specific marker genes. Neurofilament 200 kDa (*NF200*), an axonal structural protein, is involved in maintaining axonal mechanical stability; growth-associated protein 43 (*GAP43*) mediates the dynamic extension of growth cones; and neuron-specific β-tubulin III (*Tubb3*) marks terminal neuronal differentiation. In the experimental groups, the expression levels of these three key markers in the PCL/CNT + ES group were upregulated by 2.49-, 3.98-, and 1.96-fold, respectively, relative to the pure PCL group. These findings provide molecular-level evidence that the electroactive microenvironment constructed by PCL/CNT nanopatches combined with ES promotes cytoskeletal extension and synaptic maturation of differentiating neurons.

In a full-thickness skin defect model in SD rats, the PCL/CNT electroactive nanofilm combined with exogenous electrical stimulation significantly accelerated wound healing and improved the quality of tissue repair. Macroscopic observation revealed that the combined treatment group exhibited more rapid wound contraction in the early postoperative period, with a marked reduction in wound size by day 7 and nearly complete wound closure with well-filled neotissue by day 14. In contrast, groups without applied electrical stimulation still presented unhealed areas. Histological analysis using hematoxylin and eosin (HE) staining further untangled the differences in the extent of repair among groups: by postoperative day 7, the combined group had already formed thick and dense granulation tissue with epidermal coverage, whereas the control group and the group without electrical stimulation showed sparse and thin granulation tissue with incomplete epidermal coverage. By day 14, the combined group demonstrated continuous and complete epidermal coverage, well-reconstructed dermis, abundant fibroblasts, and densely arranged collagen fibers. In comparison, the other groups still exhibited epidermal defects and wider scar bands. Quantitative analysis also confirmed that the scar width in the combined group was significantly smaller than that in other groups, while the granulation tissue thickness was greater, indicating superior tissue repair quality. Masson staining results similarly demonstrated that the combined group had abundant and orderly deposition of newly synthesized collagen fibers within the wound, with a collagen-positive area significantly higher than that of the control group. This suggests that electrical stimulation combined with a conductive scaffold effectively promoted collagen synthesis and remodeling, thereby enhancing the strength and stability of the neotissue.

Notably, peripheral nerve regeneration within the cutaneous wound bed was markedly enhanced in the PCL/CNT + ES combined group. *NF200*-based immunofluorescence staining revealed that in the PCL/CNT + ES group, numerous bright *NF200*-positive peripheral nerve fibers penetrated and distributed along the newly formed granulation tissue, forming an interconnected neural fiber network. In contrast, only sparse and scattered *NF200*-positive signals were detected in the pure PCL and PCL/CNT groups (without ES intervention). These findings demonstrate that the PCL/CNT composite nanofibrous nanopatches combined with exogenous ES not only promote efficient cutaneous tissue repair but also significantly augment peripheral nerve fiber regeneration in the injured area—thereby laying a solid foundation for the recovery of cutaneous sensory function and the improvement of wound healing quality.

Exogenous electrical stimulation (ES) modulates the local regenerative niche by recapitulating the physiological endogenous electric field. On one hand, electrical signals can induce directional migration and enhance proliferation of wound repair-associated fibroblasts, epithelial cells, and other cell populations toward the cutaneous wound site, thereby accelerating wound closure and re-epithelialization (Li et al., 2012; Ud-Din, 2015). Concurrently, ES may augment local angiogenesis and nutrient perfusion at the wound bed, promoting granulation tissue maturation and ordered collagen deposition (Ud-Din, 2015). On the other hand, the electroactive microenvironment constructed by ES and electroactive nanopatches can modulate the inflammatory microenvironment, facilitating the polarization of macrophages toward a pro-reparative phenotype, alleviating tissue damage induced by excessive inflammatory responses, and creating favorable conditions for cutaneous tissue and peripheral nerve regeneration. Furthermore, this synergistic dual-modal “topological structure–electrical stimulation” strategy transduces exogenous ES into directional bioelectrical cues, providing both physical (topological) and electrical signals to guide peripheral axonal outgrowth. Finally, pathological assessment of major visceral organs (heart, liver, spleen, lung, and kidney) showed no abnormal pathological changes or toxic lesions, confirming that PCL/CNT composite nanofibrous nanopatches combined with ES exhibit excellent *in vivo* biocompatibility and systemic biosafety. Collectively, the animal experimental results fully confirm the synergistic pro-healing and pro-neuroregenerative effects of PCL/CNT composite nanofibrous nanopatches combined with exogenous ES. This approach not only accelerates cutaneous wound closure but also enhances the structural integrity and functional recovery of the newly formed tissue, providing a promising therapeutic approach for the clinical repair of complex cutaneous defects and the restoration of peripheral sensory function in the future.

## 5 Conclusion

A PCL/CNT composite nanofibrous electroactive nanopatch was successfully fabricated in the present study. By integrating exogenous electrical stimulation (ES), we constructed a synergistic dual-modal “topological structure–electrical stimulation” therapeutic strategy. This strategy not only delivers physical topological guidance and electrochemical signal modulation for axonal outgrowth but also facilitates neuronal functional remodeling via regulating the expression of neural differentiation-related genes (NF200, GAP43, and Tubb3). Furthermore, the PCL/CNT composite nanofibrous nanopatches exhibited excellent biocompatibility *in vitro* cellular experiments, validating its promising safety profile for clinical translation. In in vivo animal models of full-thickness cutaneous defects, this strategy significantly accelerated wound closure, promoted orderly tissue remodeling, and augmented peripheral nerve regeneration. Collectively, this synergistic multimodal strategy offers a novel theoretical foundation and technical approach for tissue engineering, and is anticipated to propel cutaneous wound healing toward a more efficient, precise, and high-quality paradigm.

## Data Availability

The original contributions presented in the study are included in the article, further inquiries can be directed to the corresponding authors.
